# Thermochemistry and photochemistry of spiroketals derived from indan-2-one: Stepwise processes versus coarctate fragmentations

**DOI:** 10.3762/bjoc.9.191

**Published:** 2013-08-15

**Authors:** Götz Bucher, Gernot Heitmann, Rainer Herges

**Affiliations:** 1 Lehrstuhl für Organische Chemie II, Ruhr-Universität Bochum, Universitätsstr. 150, D-44801 Bochum, Germany; 2 WestCHEM, School of Chemistry, University of Glasgow, Joseph-Black-Building, University Avenue, Glasgow G12 8QQ, United Kingdom; 3 Otto-Diels-Institut für Organische Chemie, Universität Kiel, Otto-Hahn-Platz 4, D-24098 Kiel, Germany

**Keywords:** coarctate reaction, fragmentation, matrix isolation, photolysis, pyrolysis, spiroketal

## Abstract

Coarctate reactions are defined as reactions that include atoms at which two bonds are made and two bonds are broken simultaneously. In the pursuit of the discovery of new coarctate reactions we investigate the fragmentation reactions of cyclic ketals. Three ketals with different ring sizes derived from indan-2-one were decomposed by photolysis and pyrolysis. Particularly clean is the photolysis of the indan-2-one ketal **1**, which gives *o*-quinodimethane, carbon dioxide and ethylene. The mechanism formally corresponds to a photochemically allowed coarctate fragmentation. Pyrolysis of the five-ring ketal yields a number of products. This is in agreement with the fact that coarctate fragmentation observed upon irradiation would be thermochemically forbidden, although this exclusion principle does not hold for chelotropic reactions. In contrast, fragmentation of the seven-ring ketal **3** is thermochemically allowed and photochemically forbidden. Upon pyrolysis of **3** several products were isolated that could be explained by a coarctate fragmentation. However, the reaction is less clean and stepwise mechanisms may compete.

## Introduction

**Figure 1:**
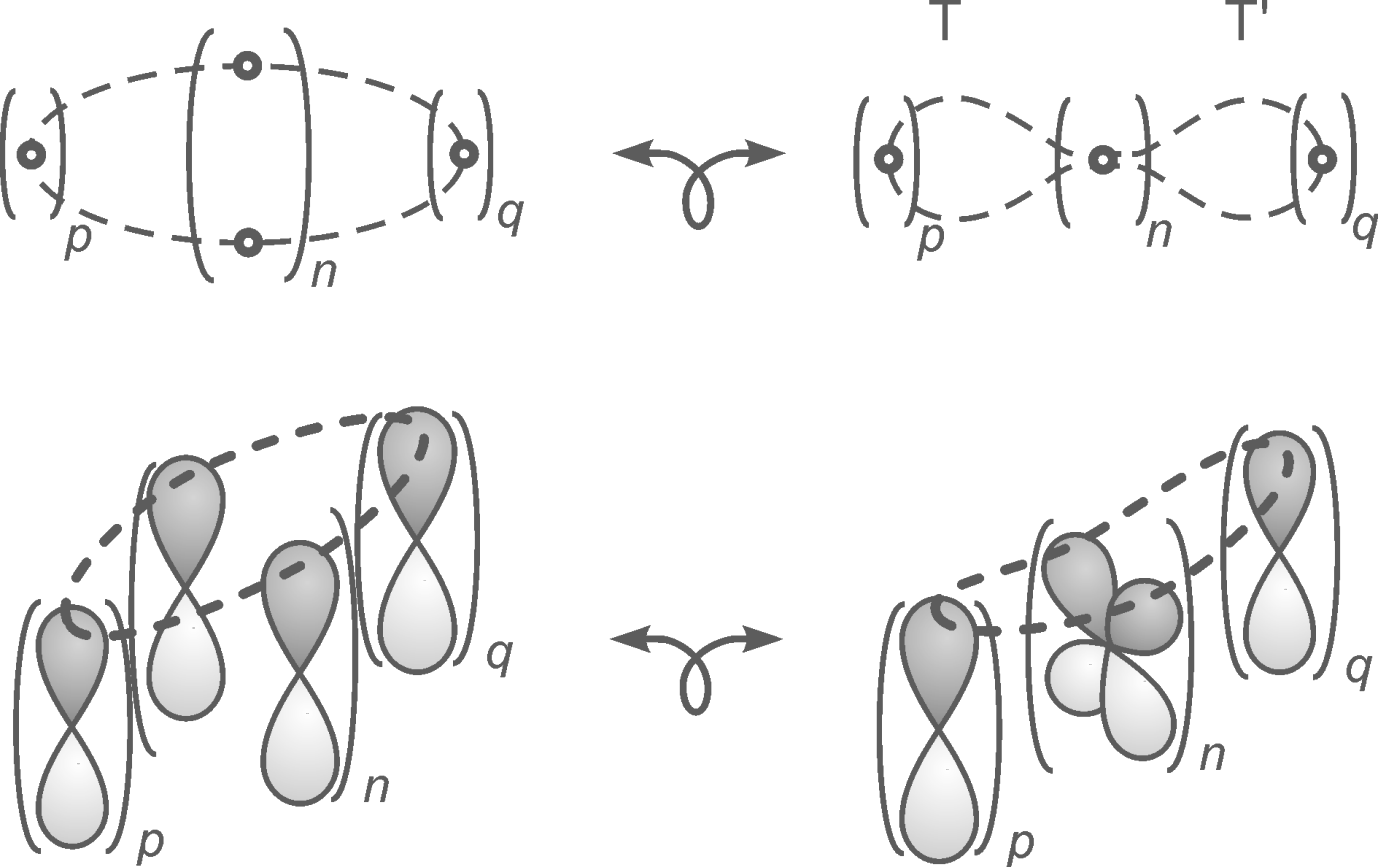
Formal, topological approach to derive coarctate reactions from pericyclic reactions; *p*, *q*: number of atoms or basis orbitals in the terminator groups T and T’; *n*: number of orthogonal pairs of basis orbitals in the transition state of coarctate reactions.

Pericyclic reactions, according to the original definition, are characterized by a cyclic array of bond making and bond breaking [1–3]. At each atom, involved in the reaction, one bond is made and one bond is broken. However, there are a number of reactions that include a linear system of atoms, or at least one atom, at which *two* bonds are made and *two* bonds broken simultaneously. Nevertheless, their transition states exhibit a cyclic overlap of basis orbitals. The orbital basis can be derived from the orbital basis of pericyclic transition states by constriction (coarctation, Figure 1). Hence, these reactions have been coined “coarctate” reactions [4–5].

**Figure 2:**
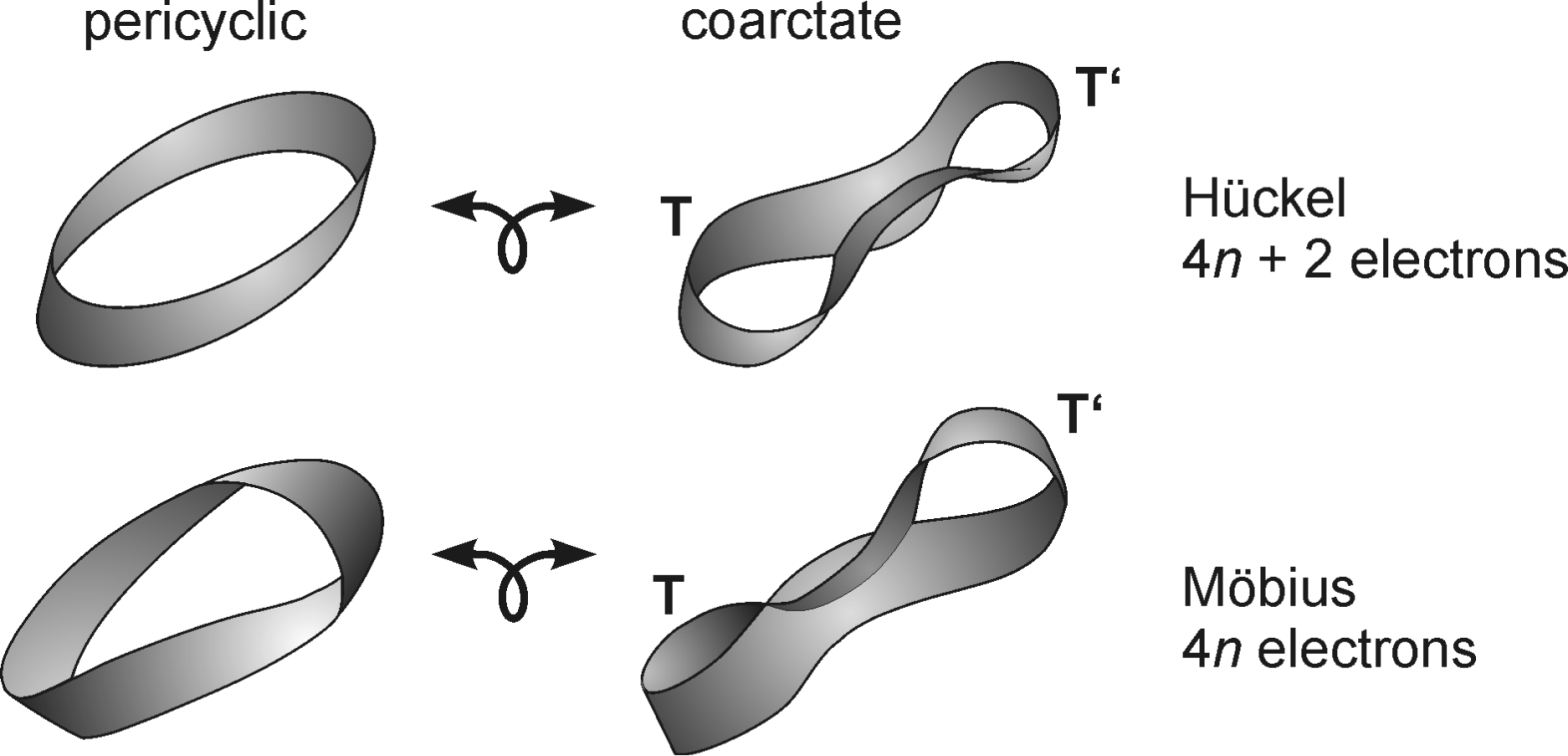
Stereochemistry of coarctate reactions derived from a Hückel (top) and a Möbius band (bottom). The terminator loops T and T’ are coplanar in the coarctate Hückel system and orthogonal in the coarctate Möbius transition state.

Similar to pericyclic reactions [6], rules were derived to predict their stereochemistry, and whether they would be thermochemically or photochemically allowed. The atom (or the linear system of atoms) at which two bonds are made and broken, each contribute two basis orbitals to the transition state (Figure 1, bottom, right). A cyclic array of orbitals is attained if the linear system of orbital overlap at each end is bound by terminating groups, e.g. a lone pair, or two atoms to form a three-ring, or four atoms to a five-ring, etc. Similar to pericyclic reactions, thermochemical coarctate reactions proceed via Hückel transition states, if the number of delocalized electrons in the transition state is 4*n* + 2, and they exhibit Möbius transition states with 4*n* electrons. If in a formal, topological transformation a closed ribbon is transformed into a coarctate band, the two loops T and T’ that are formed are coplanar. The analogous transformation of a Möbius ribbon leads to a band whose loops are orthogonal with respect to each other (Figure 2).

**Scheme 1:**
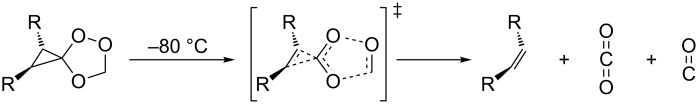
Coarctate fragmentation of the spiroozonide derived from methylenecyclopropane.

Following the above principle we developed a number of novel coarctate reactions [7–11], several of which provide synthetic access to a broad range of heterocycles [12–21]. Synthetically probably less useful, but suitable to check the coarctate stereochemical rules, is a peculiar fragmentation reaction that we discovered 15 years ago (Scheme 1) [9–10].

**Scheme 2:**
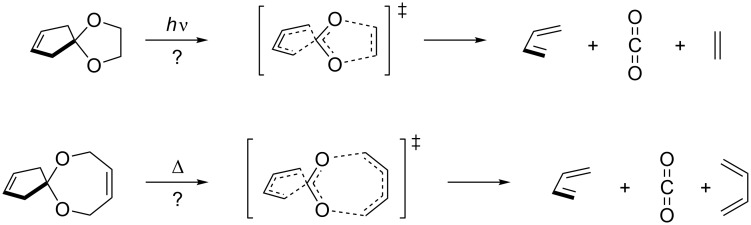
Photochemically and thermally allowed coarctate fragmentations of spiroketals.

The reaction proceeds spontaneously at temperatures below −80 °C. Quantum chemical calculations of the parent reaction predict an activation barrier of 11.3 kcal/mol and a concerted mechanism. This is in agreement with the stereochemical rule that a coarctate reaction with eight (4*n*) electrons should proceed via a Möbius transition state, with the two terminating groups orthogonal with respect to each other. The orthogonal arrangement is provided by the spiro connection of the three- and the five-membered rings. Following these rules, a Möbius type coarctate fragmentation with two five-ring terminators (10 electrons) should proceed as a photochemical reaction, and a corresponding fragmentation with a seven- and a five-ring (12 electrons) should be thermochemically activated (Scheme 2) [22].

**Scheme 3:**
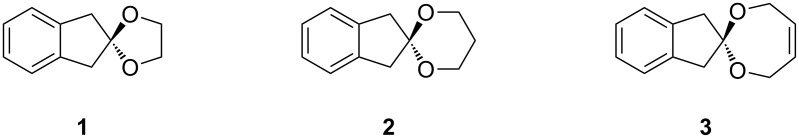
Precursors used in this study.

To test the above hypothesis, we now investigate the thermochemistry and the photochemistry of the ketals **1** and **3**, derived from indan-2-one and ethylene glycol, and *cis*-2-butene-1,4-diol (Scheme 3). The ketal **2**, derived from 1,3-propanediol, was chosen as a reference system that cannot undergo a coarctate fragmentation.

## Results

**Figure 3:**
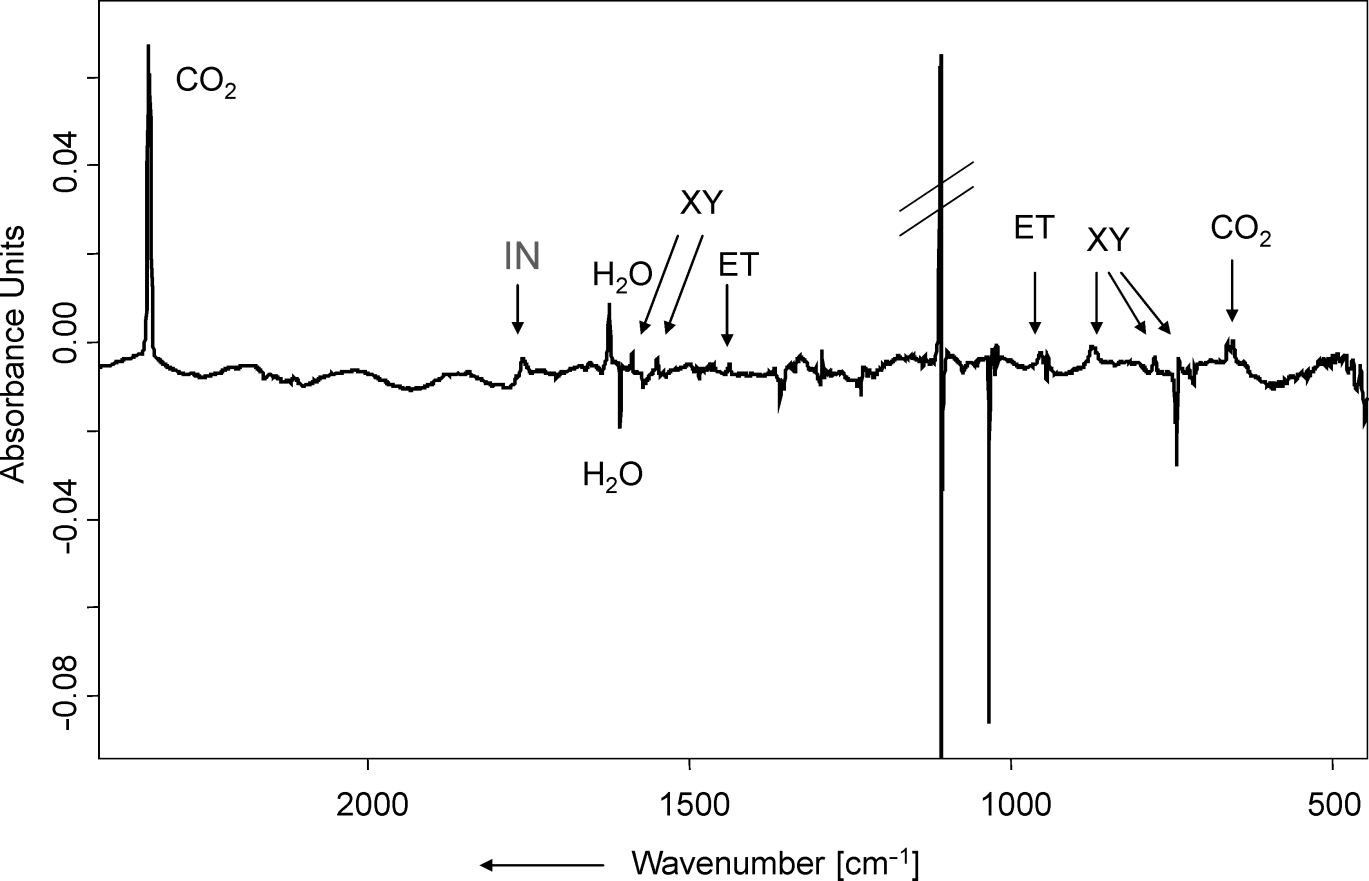
Difference infrared spectrum, showing the changes in the IR spectrum after photolysis (λ_exc_ = 254 nm, 20 min) of **1** in Ar matrix. Except for the water band, all IR bands pointing downwards belong to **1**. Product bands pointing upwards are labelled according to their assignment (**XY** = *o*-xylylene, **ET** = ethylene, **IN** = indan-2-one). The intense band pointing upwards at 1109 cm^−1^ is an artefact due to a subtraction error of a very intense precursor band.

**Photolysis and pyrolysis of the ketals.** Photolysis (λ_exc_ = 254 nm, Hg low-pressure lamp) of indan-2-one ethylene ketal (**1**), matrix-isolated in Ar at 10 K, leads to the formation of CO_2_ (vs, ν = 2342.1 cm^−1^), *o*-xylylene (**XY**, ν = 1550.4, 1470.8, 1467.4, 873.1, 776.2, 738.7 cm^−1^) [23], ethylene (**ET**, ν = 1438.1, 953.8 cm^−1^) and indan-2-one (**IN**, ν = 1761.0 cm^−1^). Some weak product bands could not be assigned. A difference IR spectrum (product bands at a very early stage of the photolysis minus precursor bands) is given in Figure 3. Figure 3 clearly shows that at least at this early stage of photolysis, practically no CO (ν = 2138.4 cm^−1^) is formed. At a later stage of photolysis (20 h, λ = 254 nm), CO is also detected, and the relative integrals of the CO_2_ and CO bands yield an estimated ratio of CO_2_ and CO of 2.5:1. It is noted, however, that the formation of CO may well be due to photolysis of CO_2_ due to hard UV radiation (λ = 185 nm) also emitted by the Hg low-pressure lamp used [24].

**Figure 4:**
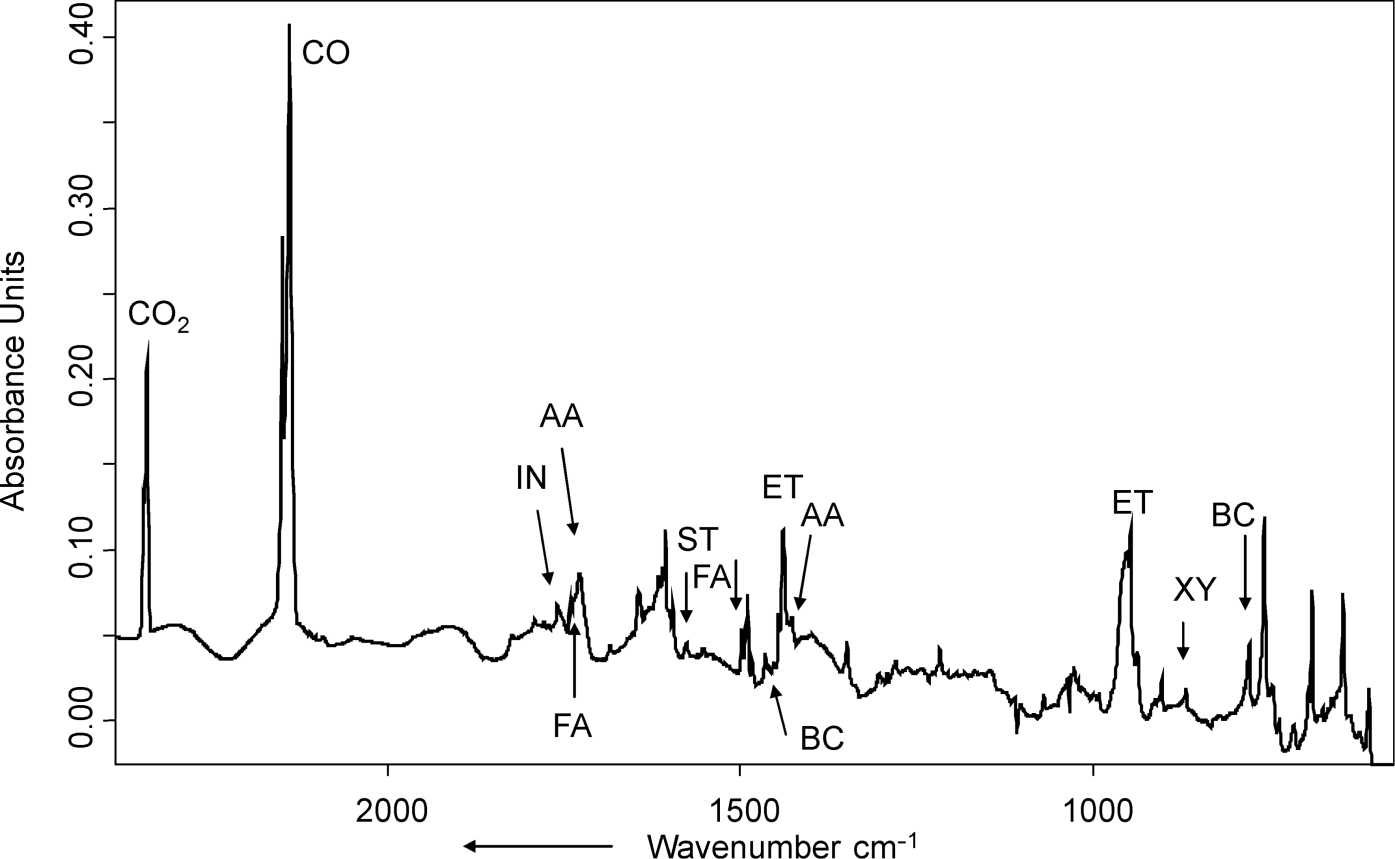
Infrared spectrum obtained upon FVP of **1** at *T* = 1143 K and trapping the pyrolysate in solid argon at *T* = 10 K.

In contrast to the very clean photochemistry of **1**, flash vacuum pyrolysis FVP (*T* = 870 °C) of **1**, followed by trapping of the reaction products in solid argon, yielded a variety of products whose identity could only partially be elucidated. The ratio of CO_2_/CO being formed in the pyrolysis reaction was different from the photochemical decomposition of **1**. Based on the integrals of the CO_2_ and CO bands, it can be estimated as 1:27. Figure 4 shows an infrared spectrum of the pyrolysis products.

The organic products include formaldehyde (**FA**), acetaldehyde (**AA**), ethene (**ET**), *o*-xylylene (**XY**), benzocyclobutene (**BC**), styrene (**ST**), and indan-2-one (**IN**). Some peaks could not be assigned. The assignment of the pyrolysis products is based on a comparison with literature data (**XY**) [21], as well as reference spectra of authentic samples (**ET**, **BC**, **IN**, **AA**, **FA**, **ST**). By calibrating IR band integrals to calculated (B3LYP/6-31G(d,p)) IR band intensities of selected bands, a crude measure of product ratios could be obtained. Relative to [CO_2_] = 1.0, the concentrations of the other pyrolysis products are as follows: [CO] = 27.2, [**IN**] = 3.8, [**ET**] = 15.9, [**BC**] = 11.4, [**XY**] = 1.3, [**AA**] = 3.9 [25]. Formaldehyde is formed as a minor product only.

**Figure 5:**
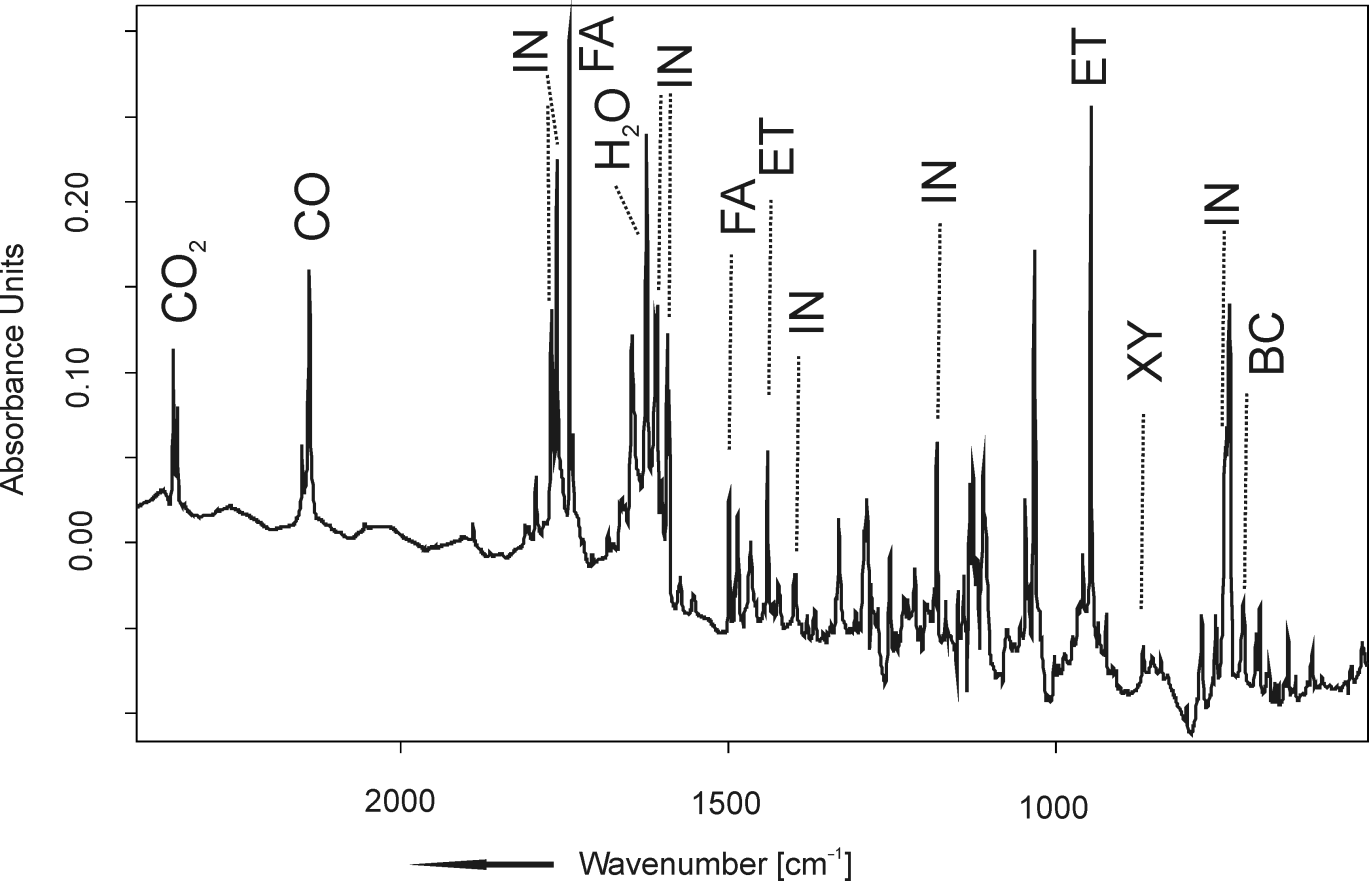
Infrared spectrum obtained upon FVP of **2** at *T* = 963 K and trapping the pyrolysate in solid argon at *T* = 10 K.

The photochemistry of ketals **2** and **3** was investigated as well by matrix isolation spectroscopy. Unfortunately no product could be unambiguously identified. The FVP of **2** yielded the product spectrum shown in Figure 5. Again, both carbon monoxide and carbon dioxide were formed along with the organic products. Carbon monoxide was formed in large excess over carbon dioxide (CO/CO_2_ = 13.5:1). Organic products include mostly **FA**, **ET**, and **IN**, as well as **BC** and **XY**, but many peaks have to remain unassigned. Propene was not formed. Compared to the FVP of **1**, the FVP of **2** yields significantly increased amounts of 2-indanone and formaldehyde. Relative to [CO_2_] = 1.0, the concentrations of the other pyrolysis products are as follows: [CO] = 13.5, [**IN**] = 9.1, [**ET**] = 15.4, [**BC**] = 19.4, [**XY**] = 1.2, [**FA**] = 12.2.

**Figure 6:**
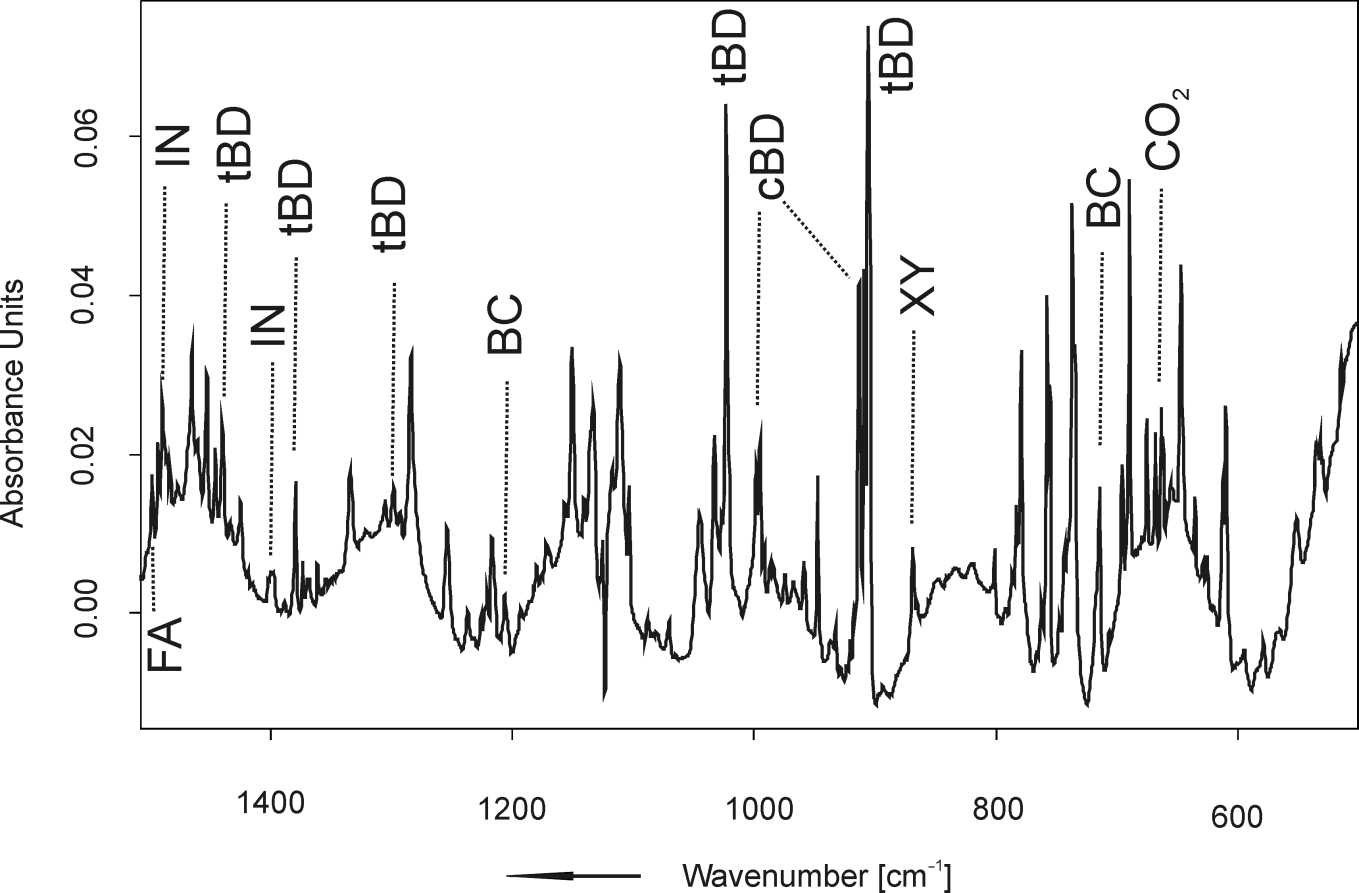
Infrared spectrum obtained upon FVP of **3** at *T* = 1043 K and trapping the pyrolysate in solid argon at *T* = 10 K.

Flash vacuum pyrolysis of indan-2-one *cis-2-*butene-1,4-diol ketal (**3**) again gave rise to a complex mixture of products (Figure 6). Among them, the two conformers of 1,3-butadiene (**tBD** and **cBD**) could be assigned based on a comparison with literature data [26]. Further products include *o*-xylylene (**XY**), benzocyclobutene (**BC**), indan-2-one (**IN**), and formaldehyde (**FA**). Again, a number of IR peaks have to remain unassigned. CO_2_ and CO are formed in a ratio of 1:2.6 in this pyrolysis reaction. Relative to [CO_2_] = 1.0, the concentrations of the pyrolysis products are as follows: [CO] = 2.6, [**IN**] = 1.0, [**tBD**] = 7.4, [**cBD**] = 1.5, [**BC**] = 6.3, [**XY**] = 0.4.

## Discussion

**Scheme 4:**
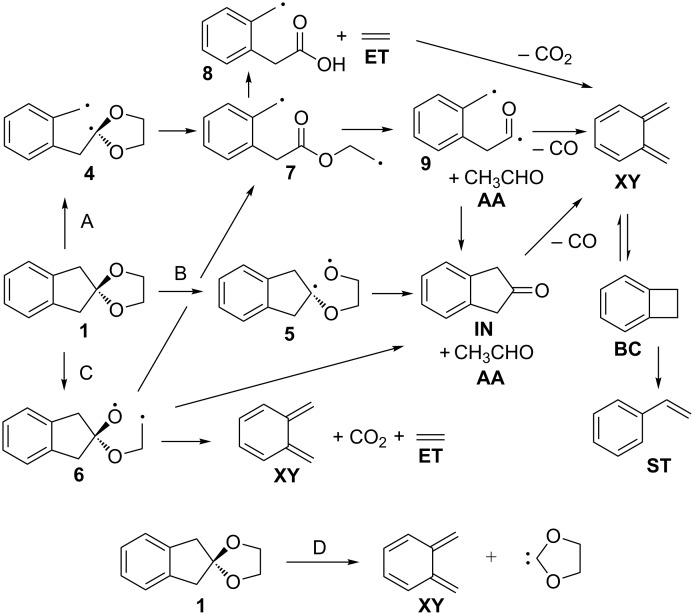
Possible fragmentation pathways in the FVP of **1**.

Flash vacuum pyrolysis of **1** yields a complex mixture, which contains a variety of fragmentation products derived from both sides of the spiroketal linkage. The relative ratio of carbon dioxide and carbon monoxide being formed (CO_2_/CO = 1:27) indicates that a coarctate fragmentation of **1** can play a minor role only, if any. The composition of the product mixture is best rationalized by a series of stepwise processes, which starts with either a C–C cleavage (pathway A, more favourable) or a C–O cleavage (pathways B or C, less favourable). In principle, a chelotropic elimination of 1,3-dioxol-2-ylidene is also conceivable (pathway D). Scheme 4 shows a possible mechanistic scenario. While it is questionable whether the biradical intermediates shown in Scheme 4 are in fact true minima or not, we note that they will be exceedingly short-lived at *T* = 1143 K in any event.

**Scheme 5:**
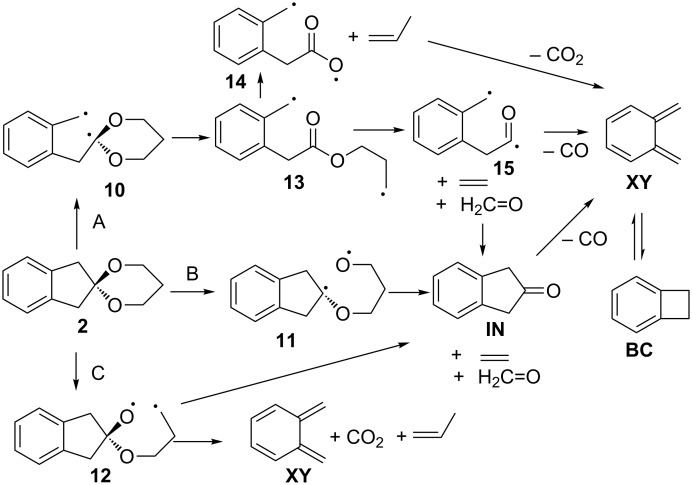
Possible fragmentation pathways in the FVP of **2**.

As the C–C bond being broken in mechanism A is significantly weaker than the C–O bonds that need to be cleaved in mechanisms B and C, pathway A is expected to be the most facile decay mechanism for **1**. The primarily formed benzyl-dialkoxymethyl biradical **4** should undergo a very facile ring-opening reaction to yield an ester biradical **7**, which can either cleave into ethylene, carbon dioxide and *o*-xylylene (**XY**), or eliminate acetaldehyde (**AA**) to yield an acyl-benzyl diradical **9** [27]. The latter can then either undergo ring closure to form indan-2-one (**IN**), or decarbonylate to give *o*-xylylene (**XY**). The equilibrium of **XY** and benzocyclobutene (**BC**) is established in the literature [28], as well as the formation of styrene **ST** from **BC** [29]. An alternative mechanism, the chelotropic elimination of 1,3-dioxolan-2-ylidene is not likely. This carbene has been generated from a norbornadiene spiro ketal, and it cleanly fragmented into CO_2_ and ethylene [30]. Theoretical calculations support the low barrier for fragmentation [31]. We explain the different reaction behaviour of our spiroketal **1** by the fact that two energetically unfavourable products would have to be formed (a quinodimethane and a carbene), whereas the fragmentation of the norbornadiene ketal gives benzene and a carbene. The mechanism for the thermal decomposition of **2** is likely to be similar. The high yield of formaldehyde in the pyrolysis of **2** is readily explained by the fact that the ester biradical **13** formed can lose one equivalent of ethene and formaldehyde to yield the acyl-benzyl type biradical **15** (Scheme 5).

**Scheme 6:**
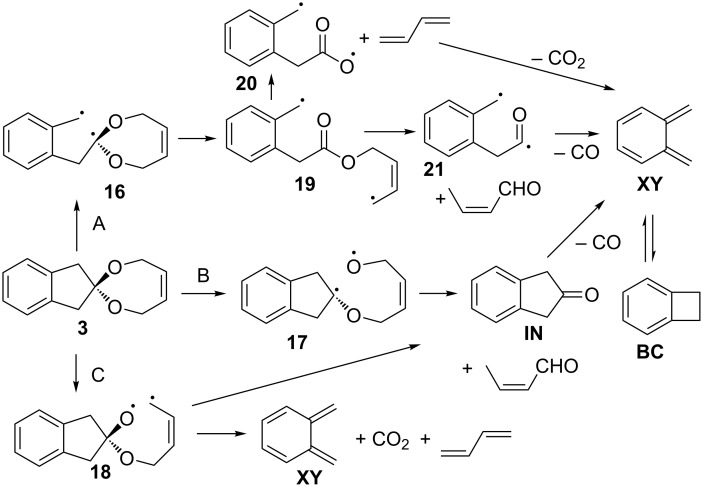
Possible fragmentation pathways in the FVP of **3**.

In the FVP of **1** and **2**, CO is formed in large excess over CO_2_. This excess is far less pronounced in the FVP of **3**. This could possibly indicate that a coarctate fragmentation of **3** (which would be a concerted version of pathway C in Scheme 3) could possibly also contribute to the product distribution (Scheme 6). A chelotropic reaction forming 4,7-dihydro-1,3-dioxepine-2-ylidene as discussed in the fragmentation of 5-ring spiroketal **1** cannot be excluded. It is known that the sulfur analogue 4,7-dihydro-1,3-dithiepine-2-yldidene cleanly fragments into carbon disulfide and butadiene [32]; however, 4,7-dihydro-1,3-dioxepine-2-ylidene does not give carbon dioxide and butadiene [33].

## Conclusion

In agreement with predictions, spiroketals derived from indan-2-one undergo photochemical coarctate fragmentation, if both terminators are 5-membered rings, and thermal coarctate fragmentation, if both a 5-ring and a 7-ring terminator are present. In the latter case, the experimental evidence suggests that the thermal coarctate fragmentation competes with stepwise processes.

## Experimental

**General:** Matrix-isolation experiments were performed using standard matrix-isolation techniques [34]. For sample deposition, the slow-spray-on technique was used. Sample temperatures for deposition were ambient temperature (**1**), ca. 40 °C (**2**), and ca. 60 °C (**3**). The argon used was of 99.999% purity. In pyrolysis experiments, the length of the pyrolysis zone was ca. 5 cm. Reference IR spectra of benzocyclobutene, styrene, indan-2-one, acetaldehyde, formaldehyde, ethene and propene in Ar matrices were independently measured. IR spectra were recorded with a resolution of 0.5 cm^−1^. The outer matrix window used for photolysis was from Suprasil quartz specified for transmission down to λ = 190 nm.

Ketal **1** was synthesized according to a published procedure [35]. Ketals **2** and **3** were prepared analogously, starting from indan-2-one and propane-1,3-diol and *cis*-2-butene-1,4-diol, respectively.

**Indan-2-one ethylene ketal (1):** IR (Ar, 10 K) ν: 3109.2 (vw), 3084.4 (vw), 3057.7 (vw), 3034.8 (w), 2993.3 (w), 2989.6 (w), 2962.3 (w), 2928.9 (vw), 2898.5 (w), 2894.8 (w), 2878.1 (w), 2834.1 (vw), 1598.6 (vw), 1485.5 (m), 1472.5 (vw), 1465.1 (vw), 1421.1 (vw), 1342.4 (vw), 1331.9 (m), 1305.9 (vw), 1292.9 (s), 1233.4 (m), 1222.9 (w), 1200.0 (w), 1160.9 (vw), 1136.8 (m), 1110.3 (vs), 1074.2 (w), 1051.9 (vw), 1033.3 (s), 1027.8 (m), 1019.7 (w), 944.4 (w), 867.0 (vw), 785.9 (vw), 741.7 (s), 715.7 (m), 597.7 (vw), 590.5 (vw), 536.8 (vw) cm^−1^.

**Synthesis of 2:** Indan-2-one propane-1,3-diol ketal (**2**) was prepared as described for the synthesis of **1**, with the exception of the use of toluene rather than benzene as solvent. Indan-2-one (2.0 g, 0.015 mol) and 1,3-propanediol (1.4 g, 0.018 mol) were heated under reflux in 100 mL toluene together with 20 mg *p*-toluenesulfonic acid. The mixture was heated under reflux for 12 h, during which time the water formed was distilled off as an azeotrope with toluene. The toluenic solution was then washed twice with aq NaHCO_3_ and once with water. After drying over anhydrous Na_2_SO_4_, the toluene was removed on a rotary evaporator. Purification of the crude product thus obtained was achieved by distillation in high vacuum. Yield 1.2 g (42%) after distillation. bp 93–97 °C (0.01 mbar); mp 44 °C; ^1^H NMR (CDCl_3_, 400 MHz) δ 7.14 (m, 4H), 3.97 (t, *J* = 5.5 Hz, 4H), 3.28 (s, 4H), 1.78 (m, 2H) ppm; ^13^C NMR (CDCl_3_, 100 MHz) δ 139.68, 126.65, 124.71, 109.23, 61.52, 42.54, 25.56 ppm; IR (Ar, 10 K) ν: 3111.7 (vw), 3084.8 (vw), 3076.7 (vw), 3060.8 (vw), 3050.1 (w), 3039.8 (w), 3035.7 (w), 2993.7 (w), 2983.8 (m), 2980.1 (m), 2972.7 (m), 2962.7 (m), 2949.8 (m), 2943.1 (m), 2930.7 (m), 2908.2 (w), 2899.4 (w), 2891.9 (w), 2884.6 (m), 2879.0 (m), 2876.1 (m), 2858.9 (m), 2849.9 (w), 2727.6 (vw), 2717.7 (vw), 1620.4 (w), 1612.1 (vw), 1592.5 (vw), 1572.9 (vw), 1488.3 (m), 1477.1 (w), 1464.8 (w), 1433.4 (w), 1425.9 (w), 1381.5 (w), 1369.9 (w), 1333.7 (m), 1305.7 (vw), 1298.5 (w), 1284.5 (vs), 1254.1 (m), 1237.4 (w), 1215.2 (w), 1170.3 (vw), 1150.9 (vs), 1135.6 (vs), 1133.0 (vs), 1117.7 (s), 1111.0 (vs), 1081.2 (w), 1045.9 (s), 1032.9 (s), 1024.0 (m), 966.3 (w), 937.6 (w), 868.8 (w), 734.8 (s), 678.9 (vw), 607.2 (vw), 594.3 (vw), 555.4 (vw) cm^−1^; EIMS *m*/*z*: M^+^ 190 (100), 176, 161, 132 (68), 104 (80), 91, 77, 51; Anal. calcd for C_12_H_14_O_2_: C, 75.8; H, 7.4; found: C, 75.3; H, 7.2.

Indan-2-one cis-2-butene-1,4-diol ketal (**3**) was prepared analogously. Due to the limited thermal stability of **3**, benzene had to be used as solvent, and the product could not be distilled. Instead, a sample of the solid dark brown crude product was purified by sublimation in ultra-high vacuum (10^−6^ mbar), using matrix-isolation equipment. Colourless crystals, mp 78 °C; ^1^H NMR (CDCl_3_, 400 MHz) δ 7.16 (m, 4H), 5.73 (t, *J* = 1.5 Hz, 2H), 4.31 (d, *J* = 1.5 Hz, 4H), 3.25 (s, 4H) ppm; ^13^C NMR (CDCl_3_, 100 MHz) δ 139.84, 129.53, 126.63, 124.64, 113.31, 62.93, 44.07 ppm; IR (Ar, 10 K) ν: 3078.6 (vw), 3058.5 (vw), 3046.1 (w), 2986.4 (vw), 2966.8 (vw), 2952.9 (w), 2949.1 (w), 2945.8 (w), 2926.2 (w), 2920.9 (w), 2912.3 (w), 2908.0 (w), 2865.0 (w), 2838.8 (vw), 2718.3 (vw), 1622.9 (w), 1612.3 (vw), 1607.9 (vw), 1592.9 (vw), 1589.4 (vw), 1573.0 (vw), 1487.9 (m), 1465.8 (w), 1449.2 (w), 1425.2 (w), 1390.2 (w), 1363.3 (w), 1330.3 (m), 1306.4 (w), 1284.9 (s), 1227.6 (m), 1220.9 (w), 1201.7 (m), 1167.6 (w), 1155.8 (w), 1123.8 (vs), 1115.4 (w), 1102.2 (vw), 1090.2 (s), 1078.3 (m), 1044.6 (s), 1026.9 (m), 1009.9 (m), 951.9 (vw), 946.6 (vw), 920.8 (vw), 879.4 (vw), 872.8 (vw), 817.4 (vw), 732.5 (s), 684.8 (vw), 667.8 (vw), 641.8 (m), 619.4 (m), 617.4 (m), 595.8 (w), 559.1 (vw), 527.5 (vw) cm^−1^; EIMS *m*/*z*: M^+^ 202 (55), 176, 161, 149, 148, 147, 132, 104 (100), 91, 78, 54 (95), 51, 39; Anal. calcd for for C_13_H_14_O_2_: C, 77.2; H, 7.0; found: C, 77.5; H, 7.0; HRMS–ESI (*m*/*z*): [M]^+^ calcd for C_13_H_14_O_2_Na, 225.0898; found, 225.0891.
